# Meta-Analysis of the Factors Influencing the Restoration of Spontaneous Circulation After Cardiopulmonary Resuscitation

**DOI:** 10.3389/fphys.2022.834352

**Published:** 2022-03-08

**Authors:** Hui-Ru An, Yi-Ran Han, Tian-Hao Wang, Fei Chi, Yu Meng, Chun-Yan Zhang, Jian-Qin Liang, Xiang-Lan Li

**Affiliations:** ^1^Department of Tuberculosis, Senior Department of Tuberculosis, The Eighth Medical Center of PLA General Hospital, Beijing, China; ^2^Emergency Department, The Eighth Medical Center of PLA General Hospital, Beijing, China; ^3^Emergency Department, Hebei Chest Hospital, Shijiazhuang City, China

**Keywords:** cardiopulmonary resuscitation, return of spontaneous circulation, influencing factors, meta-analysis, cardiopulmonary arrest

## Abstract

**Objective:**

This study aimed to systematically evaluate the factors influencing the restoration of spontaneous circulation (ROSC) after cardiopulmonary arrest (CA).

**Methods:**

Relevant papers on the factors influencing the ROSC in patients with CA were retrieved from PubMed, Embase, Cochrane Library, China Biology Medicine disk, China National Knowledge Infrastructure, Wanfang, and VIP databases. After screening, data extraction, and quality evaluation of the papers, a meta-analysis was carried out.

**Results:**

A total of 36 papers, involving a total sample size of 2,305 cases, were included. The meta-analysis revealed that the location and time of onset of CA, the type of cardiac rhythm at first monitoring, the start time of cardiopulmonary resuscitation (CPR), the use of electric defibrillation, and the cumulative dose of adrenaline all significantly impacted the ROSC (*p* < 0.05) and may have affected its success rate. The pH value at CA onset, combined use of adrenaline and vasopressin, CPR duration, mechanical cardiac compression use, and whether CA was caused by heart disease had no significant effect on ROSC.

**Conclusion:**

The location and time of onset of CA, the cardiac rhythm at first monitoring, the start time of CPR, the use of electric defibrillation, and the cumulative dose of adrenaline significantly impacted the ROSC.

## Introduction

Cardiopulmonary arrest (CA) is a common acute and critical illness in which the heartbeat ceases without warning and the patient suddenly stops breathing. There is a sudden loss of consciousness, respiratory arrest, or weak, sigh-like breathing, and no pulse can be felt. Cardiopulmonary resuscitation (CPR) is an important measure used to treat *CA*. Although CPR and other advanced recovery technologies are improving, the restoration of spontaneous circulation (ROSC) rate is still very low (Tang et al., 2017). This study systematically evaluated the factors influencing the ROSC in patients with CA to provide a reference point for future treatment.

## Materials and Methods

### Literature Retrieval

Relevant studies, published between 1 January 2010, and 1 January 2021, were retrieved from databases including PubMed, Embase, the China Biology Medicine disk, the China National Knowledge Infrastructure, and the Wanfang, and VIP databases. The English keywords were “cardiovascular resuscitation,” “diagnostic factors,” “cardiac arrest,” and “factors analysis.” The Chinese keywords were “心肺复苏,” “心脏骤停,” “心搏骤停,” and “预后.” In addition, the references of all the included papers were retrieved to identify more relevant studies. All documents were entered onto the Endnote reference management software. Two researchers independently screened the papers to evaluate the eligibility of the studies and were blinded to each other’s decisions. Standard data extraction tables were used to extract data and to crosscheck the results to minimize bias and randomization errors in the data analysis.

### Inclusion and Exclusion Criteria

Inclusion criteria: (1) All the included patients were diagnosed with CA in emergency departments; (2) all patients underwent CPR; (3) all studies were of a randomized controlled, case–control, or cohort design; and (4) complete data were available.

Exclusion criteria: (1) The full text or quantitative extraction of indicators was not available; (2) the paper was a review, a case report, an animal study, or nursing-related or *in vitro* research; (3) the paper was a duplicate or of a low quality; and (4) the language was not Chinese or English.

### Data Extraction and Quality Evaluation

For each paper, the first author, year of publication, country or region of publication, sample size, gender, age, clinical indicators, and other patient-related data were extracted. The quality of the cohort studies was evaluated by two researchers in strict accordance with the Newcastle–Ottawa Scale (NOS). Where there was disagreement, this was discussed by both parties or ruled upon by a third researcher.

### Statistical Analysis

The data were statistically analyzed using the statistical software STATA 11.0. Cochran’s Q test and the *I*^2^ test were used to test for heterogeneity. If *p* < 0.05 or *I*^2^ > 50%, the random effect model was adopted, and if *p* > 0.05 or *I*^2^ < 50%, the fixed-effects model was adopted. The risk ratio (RR) and a 95% CI were used to evaluate the therapeutic effect of binary variables. Standardized mean difference (SMD) or weighted mean difference (WMD) was used to analyze continuous variables according to whether the units of specific indicators were unified. The inspection level of the meta-analysis was set as *α* = 0.05, and the 95% CI was calculated for all analyses. Egger’s test was used to evaluate publication bias, while evaluation quality bias was eliminated by a sensitivity analysis.

## Results

### Characteristics of the Selected Studies

**Figure 1:**
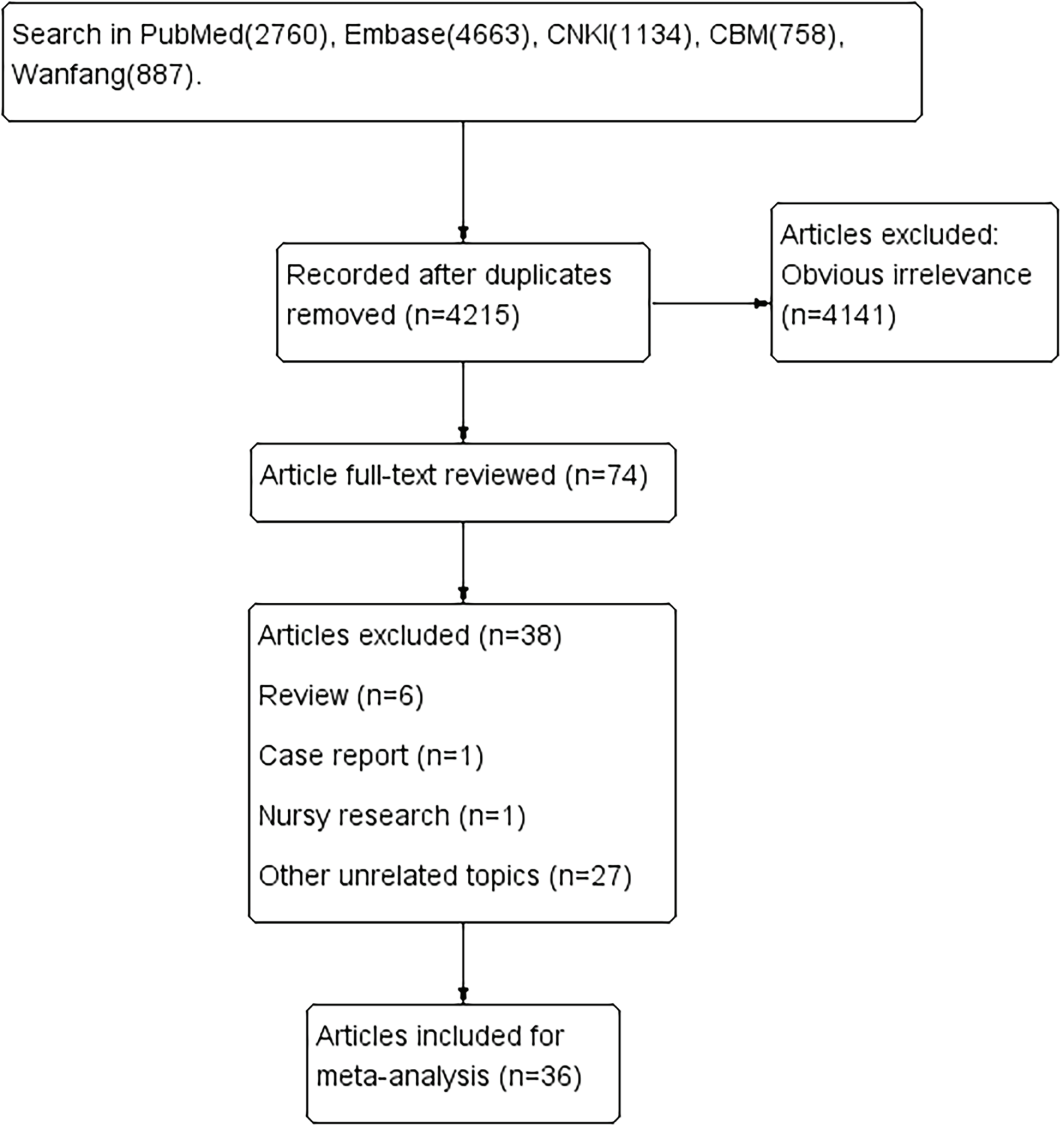
The literature retrieval flow chart.

**Figure 2:**
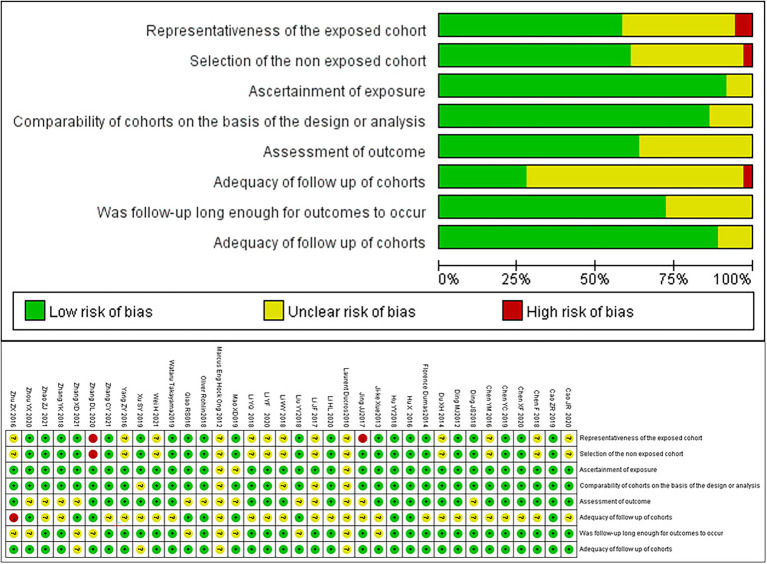
The quality evaluation of the included studies.

A total of 10,202 papers were retrieved. After excluding duplicate and irrelevant papers, a total of 36 studies, based on 2,305 patients, were included. The literature retrieval flow chart is shown in Figure 1. The characteristics of the included papers are shown in Table 1. The results of the evaluation of the study quality, according to the NOS scale, are shown in Figure 2.

**Table 1:** Basic situations of included literatures.

References	Sample size		Age			Gender		State	Literature type	NOS score	Outcome indicator
	Control group	Observation group	Control group	Observation group	Average age	Male	Female			
Dumas et al., 2014	1,134	422	60.3	58.3	NA	1,112	444	France	CS	8	➉
Xue et al., 2013	187	538	NA	NA	NA	519	206	China	CS	6	①②③④⑥⑦➉
Ducros et al., 2011	16	14	60 ± 4	56 ± 4	NA	25	5	France	RCT	0	➉
Ong et al., 2012	353	374	64.9	64.6	NA	505	222	Singapore	RCT	0	➉
Rohlin et al., 2018	1,639	798	NA	NA	NA	145	103	Sweden	CS	8	⑦
Takayama et al., 2019	948	306	72	67	NA	837	417	Japan	CS	8	②
Cai, 2020	39	61	72.1 ± 13.25	45.2 ± 11.23	NA	23	16	China	CS	5	③④
Ding and Xie, 2012	82	72	NA	NA	NA	98	56	China	CS	8	⑤
Qiao et al., 2016	77	71	NA	NA	NA	85	63	China	CS	5	⑤
Liu, 2018	125	75	NA	NA	NA	137	63	China	CS	7	①⑦⑧➉
Mao, 2019	147	71	51	73	NA	143	75	China	CS	5	
Zhou et al., 2020	71	27	NA	NA	NA	58	40	China	CS	6	③⑨➉
Zhang et al., 2021a	40	28	NA	NA	NA	39	29	China	CS	5	③⑤➉
Zhang et al., 2018	36	57	71.33 ± 8.25	72.45 ± 7.64	NA	51	42	China	CS	6	③⑥⑦➉
Zhang et al., 2021b	38	114	NA	NA	51.43 ± 15.29	27	11	China	CS	7	①②③④⑥➉
Zhang, 2020	100	100	70.4 ± 6.48	51.2 ± 6.11	NA	58	42	China	CS	5	
Xu et al., 2019	861	530	NA	NA	60.1 ± 18.9	821	570	China	CS	5	②③④⑦⑧➉
Jing et al., 2017	72	321	65.6 ± 17.32	65.0 ± 16.43	NA	284	109	China	CS	5	①
Li, 2017	37	21	NA	NA	45.27 ± 6.18	31	27	China	CS	3	
Li et al., 2018a	55	31	NA	NA	NA	49	37	China	CS	4	
Li, 2020	46	74	NA	NA	66.5 ± 6.6	72	48	China	CS	7	①②③④⑥⑦➉
Li and Jia, 2020	50	53	54.1 ± 10.37	53.7 ± 11.25	NA	56	47	China	CS	5	➉
Du, 2014	9	51	NA	NA	46.8 ± 9.7	37	23	China	CS	5	
Yang et al., 2016	75	223	NA	NA	NA	205	93	China	CS	5	②③④⑥➉
Hu et al., 2018	208	405	NA	NA	59.42 ± 18.61	410	203	China	CS	8	⑤⑨
Hu and Lu, 2016	129	152	NA	NA	NA	161	120	China	CS	8	②⑤
Cai et al., 2019	81	71	NA	NA	65.33 ± 14.98	96	56	China	CS	8	②⑧
Chen and Luo, 2018	48	56	NA	NA	NA	71	33	China	CS	5	②⑦➉
Chen et al., 2020	71	31	NA	NA	69.9 ± 17.7	57	45	China	CS	7	①③⑥⑦
Chen, 2019	18	64	NA	NA	56.17 ± 8.11	50	32	China	CS	7	
Chen et al., 2016	29	105	NA	NA	NA	92	42	China	CS	5	
Wei, 2021	16	104	NA	NA	60.2 ± 10.4	83	37	China	CS	5	①②③④➉
Li et al., 2018b	39	185	61.3 ± 13.4	60.3 ± 12.8	NA	140	84	China	CS	5	②➉
Zhao et al., 2021	41	41	44.89 ± 6.92	46.31 ± 7.86	NA	50	32	China	CS	6	⑨
Ding and Zhang, 2018	32	32	45.63 ± 2.58	46.85 ± 3.15	NA	37	27	China	CS	6	⑨
Zhu et al., 2016	49	49	36.4 ± 5.7	40.3 ± 3.4	NA	50	48	China	CS	4	⑨

① *Impact of CA onset location on ROSC;* ② *impact of CA onset time on ROSC;* ③ *impact of whether cardiogenic CA on ROSC;* ④ *impact of whether defibrillable rhythm on ROSC;* ⑤ *impact of pH on ROSC in patients with CA;* ⑥ *impact of CPR start time on ROSC;* ⑦ *impact of CPR duration on ROSC;* ⑧ *impact of whether electrical defibrillation on ROSC in patients with CA;* ⑨ *impact of whether CPR machine is used on ROSC in patients with CA;* ➉ *impact of cumulative dose of adrenaline on ROSC in patients with CA; and* ➉ *impact of combination of adrenaline and vasopressin on ROSC in patients with CA*.

### Meta-Analysis Results

#### Meta-Analysis of the Impact of Location and Time of Onset of CA on the ROSC

**Figure 3:**
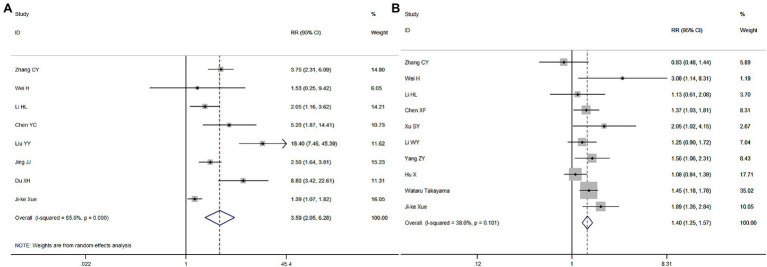
Meta-analysis forest maps of the impact of cardiopulmonary arrest (CA) onset location **(A)** and time **(B)** on the restoration of spontaneous circulation (ROSC).

Eight and 10 articles, respectively, investigated these relationships (Figures 3A,B). The meta-analysis revealed that, compared with out-of-hospital CA, the RR for the ROSC in the hospital was 3.59 (95% CI = 2.05–6.28, *p* < 0.001), and compared with CA at night, the RR for the ROSC during the day was 1.40 (95% CI = 1.25–1.57, *p* < 0.001). Therefore, both the location and time of CA are associated with the success rate of the ROSC.

#### Meta-Analysis of the Type of CA and Rhythm, and pH Value Before Treatment, on the ROSC

**Figure 4:**
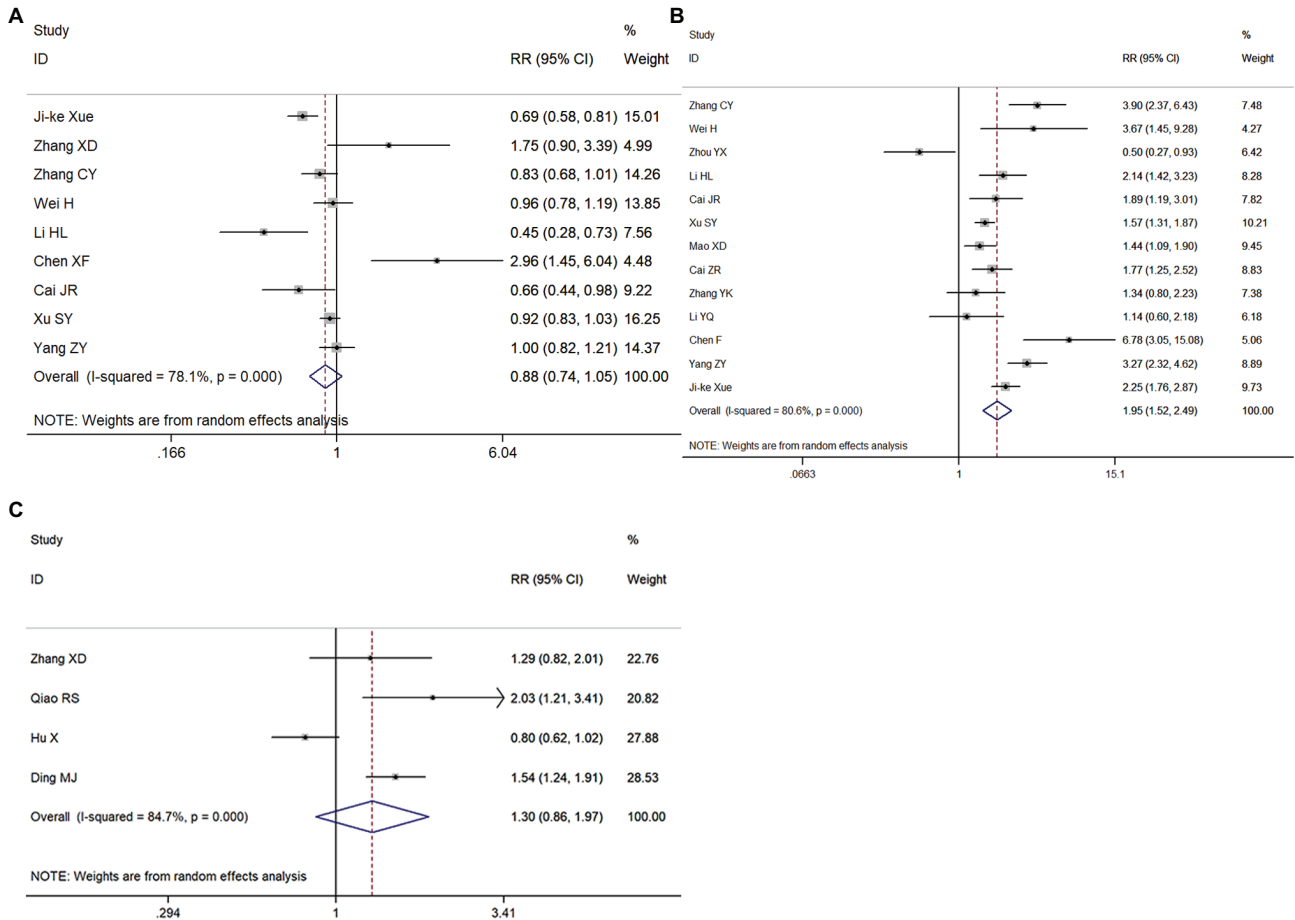
Meta-analysis forest maps of the impact of CA type **(A)**, the type of rhythm **(B),** and the pH value of patients **(C)** on the ROSC.

Nine, 13, and four papers, respectively, explored the relationships between these factors and the ROSC. Cardiac CA refers to CA that is caused by heart disease, non-cardiac CA refers to CA that is not caused by heart disease. Compared with patients with cardiac CA, the RR for the ROSC in patients with non-cardiac CA was 0.88 (95% CI = 0.74–1.05, *p* = 0.167; Figure 4A). Compared with patients whose heart rhythm was not amenable to direct current cardioversion/defibrillation, the RR for the ROSC in CA patients with shockable rhythms was 1.95 (95% CI = 1.52–2.49, *p* < 0.001; Figure 4B). Finally, the RR for the ROSC in patients with pH > 7.0 was 1.26 (95% CI = 0.75–2.11, *p* = 0.38) compared with patients with pH < 7.0 (Figure 4C). Therefore, only a shockable rhythm was related to a successful ROSC.

#### Meta-Analysis of the Effect of CPR Start Time and Duration on the ROSC

**Figure 5:**
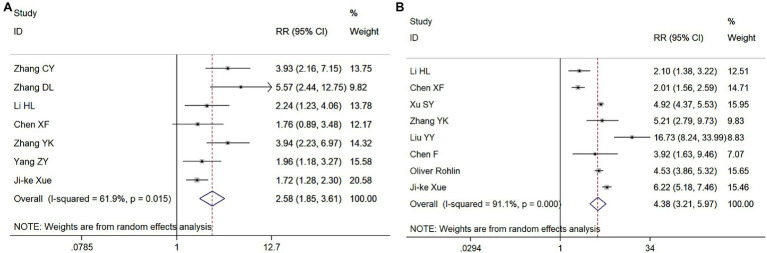
Meta-analysis forest maps of the impact of cardiopulmonary resuscitation (CPR) start time **(A)** and CPR duration **(B)** on the ROSC.

Seven and eight articles, respectively, were included in these meta-analyses. Compared with a CPR start time >5 min, the RR for the ROSC in patients with a CPR start time ≤5 min was 2.58 (95% CI = 1.85–3.61, *p* < 0.001; Figure 5A); the RR for the ROSC in patients with a CPR duration ≤15 min was 4.38 (95% CI = 3.21–5.97, *p* < 0.001), compared with a CPR duration >15 min (Figure 5B). Therefore, both the start time and the duration of CPR were significantly associated with the ROSC in patients with *CA*.

#### Meta-Analysis of the Effects of the Use of Electrical Defibrillation and CPR Machines on the ROSC

**Figure 6:**
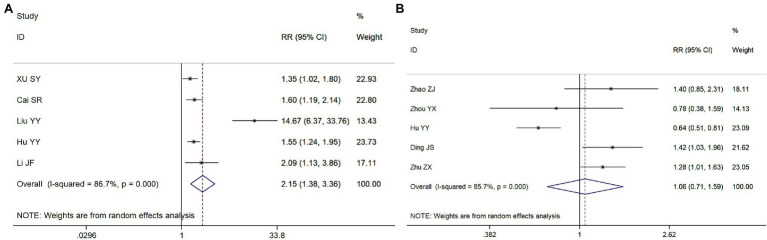
Meta-analysis forest maps of the impact of the use of electrical defibrillation **(A)** and machine chest compression systems **(B)** on the ROSC.

Five independent studies were included in the meta-analyses to study the effect of the use of electric defibrillation and CPR machines on the ROSC in patients with *CA*. Compared with patients in whom electrical defibrillation was not used, the RR for the ROSC in patients receiving electrical defibrillation was 2.15 (95% CI = 1.38–3.36, *p* = 0.01; Figure 6A). However, compared with patients receiving manual chest compression, the RR for patients on whom a CPR machine was used was 1.06 (95% CI = 0.71–1.59, *p* = 0.784; Figure 6B). Therefore, electric defibrillation therapy was positively associated with the ROSC in patients with CA, while machine chest compression systems had no significant influence on ROSC.

#### Meta-Analysis of the Effects of the Cumulative Dose of Adrenaline and the Combined Use of Adrenaline and Vasopressin on the ROSC

**Figure 7:**
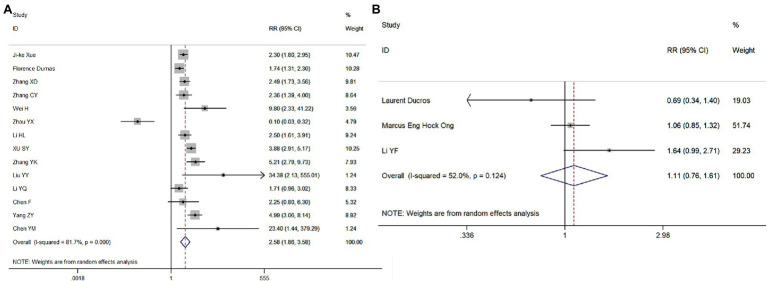
Meta-analysis forest maps of the impact of the cumulative dose of adrenaline **(A)** and the combined use of adrenaline and vasopressin **(B)** on the ROSC.

Fourteen and three studies, respectively, were included in these analyses. Compared with patients receiving >5 mg of adrenaline, the RR for the ROSC in patients receiving ≤5 mg of adrenaline was 2.58 (95% CI = 1.86–3.58, *p* < 0.001; Figure 7A), while the RR for the ROSC in patients treated with adrenaline and vasopressin was 1.08 (95% CI = 0.83–1.41, *p* = 0.564), compared to those treated with adrenaline only (Figure 7B). Therefore, the cumulative dose of adrenaline was negatively correlated with the ROSC in patients with *CA*.

### Publication Bias and Sensitivity Analysis

**Figure 8:**
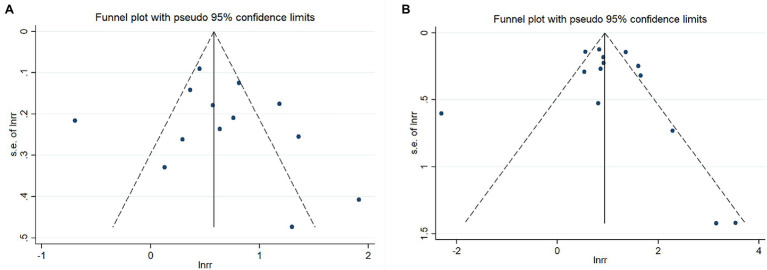
Funnel plots of defibrillation rhythm **(A)** and the cumulative dose of adrenaline **(B)**.

Egger’s tests and funnel plot analyses were carried out for 11 indexes, which revealed no publication bias in any of them (*p* > 0.05). Some of the funnel diagrams are shown in Figures 8A,B.

## Discussion

Cardiac arrest is the most critical state seen in clinical practice. Although diagnosis and treatment have progressed in recent years, the prognosis for CA is still poor. Multiple factors impact the prognosis of the ROSC in patients with CA (Neumar et al., 2015). Medical staff must assess these so that the prognosis of patients can be more accurately judged and patients can receive the correct and timely treatment. This meta-analysis revealed that the location and time of onset of CA, the type of cardiac rhythm, the start time of CPR, the use of external defibrillators defibrillators, and the cumulative dose of adrenaline significantly impacted the ROSC and may affect its success rate.

This meta-analysis revealed that the RRs for the ROSC in patients who suffered a CA during the day or in the hospital were significantly increased. Additionally, a considerable proportion of patients who suffer a CA at night or outside of the hospital may not receive CPR in time or in the correct manner. Furthermore, the ROSC in patients who received CPR within 5 min was also significantly increased. This suggests that the earlier CPR starts, the greater the likelihood of successful ROSC in patients with *CA*. Zhao reported that the smaller the rescue radius, and the shorter the CA duration, the higher the rate of successful resuscitation (Zhao, 2017). A previous study also revealed that CPR given by eyewitnesses and electrical defibrillation were both associated with a 1-year improvement in neurological function and the reduction of all-cause mortality (Kragholm Wissenberg et al., 2017). Therefore, familiarization with CPR and rapid provision of CPR will greatly improve the success rate of treatment for patients with *CA*.

Whether CA is caused by heart disease and the PH value before treatment were not related to ROSC, but electrical defibrillation was given to patients with defibrillation rhythm or CA, suggesting that electrocardiography or electrocardiogram monitoring should be performed immediately if conditions permit to facilitate timely defibrillation. When the initial heart rhythm is ventricular fibrillation or ventricular tachycardia before electric defibrillation, the heart is in the condition of weak and irregular contraction. Therefore, the CPR in patients with ventricular fibrillation or ventricular tachycardia has a better performance. Where this is not possible, patients can be treated by electric defibrillation, which increases the probability of successful ROSC.

This meta-analysis showed that CPR of a shorter duration increased the probability of the ROSC in patients with *CA*. Similarly, a cumulative dose of adrenaline was negatively correlated with the ROSC. The longer duration of CPR will lead to more serious ischemia of vital organs, such as the heart, lung, and brain. Prolonged CPR may result in irreversible damage to these vital organs and result in poor patient prognosis even if ROSC occurs. Epinephrine has a more pronounced treatment benefit when given early in the resuscitation process, especially for non-shockable cardiac arrest (Soar and Berg, 2021). It is both a beta- and alpha-adrenergic receptor agonist. Its alpha effects cause peripheral vasoconstriction, which leads to increased blood flow in the central circulation, improving perfusion of the brain and heart. Epinephrine’s beta-adrenergic effects increase heart rate, contractility, and automaticity. These potentially deleterious effects may lead to ventricular tachyarrhythmias and increased myocardial oxygen demand (Jung et al., 2018). Furthermore, the gradual accumulation of adrenaline leads to an increase in adverse reactions, such as visceral vasoconstriction, organ injury, and malignant arrhythmia.

The use of CPR machines does not increase the probability of successful ROSC compared with manual chest compression. Machine chest compression systems save on manpower (by decreasing or eliminating fatigue) and can automatically achieve the best balance between ventilation and compression. However, manual chest compression is simple to operate and does not require the use of other instruments, and it is not affected by factors such as time and location. Therefore, it is widely used in settings where a CPR machine cannot be installed. Furthermore, manual chest compression does not decrease the probability of ROSC in patients with *CA*.

This meta-analysis has some limitations. The quality of some of the papers included was low, and some of the factors were examined by only a few studies, which may have restricted the validity of the results. Therefore, the results of this meta-analysis should be treated with caution. The factors influencing the ROSC after CA must be confirmed by large-scale clinical trials to provide a practical and reliable reference basis for clinical treatment.

In summary, the location and time of onset of CA, the first monitored rhythm, the start time of CPR, the use of electric defibrillation, and the cumulative dose of adrenaline had significant effects on ROSC.

## Data Availability Statement

The original contributions presented in the study are included in the article/supplementary material; further inquiries can be directed to the corresponding authors.

## Ethics Statement

Ethical review and approval was not required for the study on human participants in accordance with the local legislation and institutional requirements. Written informed consent for participation was not required for this study in accordance with the national legislation and the institutional requirements.

## Author Contributions

H-RA, J-QL, and X-LL: conception and design of the research. Y-RH, FC, and YM: acquisition of data. Y-RH and H-RA: analysis and interpretation of the data. Y-RH and C-YZ: statistical analysis. X-LL: obtaining financing. H-RA, Y-RH, and J-QL: writing of the manuscript. T-HW and X-LL: critical revision of the manuscript for intellectual content. All authors contributed to the article and approved the submitted version.

## Conflict of Interest

The authors declare that the research was conducted in the absence of any commercial or financial relationships that could be construed as a potential conflict of interest.

## Publisher’s Note

All claims expressed in this article are solely those of the authors and do not necessarily represent those of their affiliated organizations, or those of the publisher, the editors and the reviewers. Any product that may be evaluated in this article, or claim that may be made by its manufacturer, is not guaranteed or endorsed by the publisher.
